# Is Decompressive Surgery for Cervical Spondylotic Myelopathy Effective in Patients Suffering from Concomitant Multiple Sclerosis or Parkinson’s Disease?

**DOI:** 10.3390/brainsci7040039

**Published:** 2017-04-10

**Authors:** Taylor E. Purvis, Daniel Lubelski, Thomas E. Mroz

**Affiliations:** 1 Department of Neurosurgery, Johns Hopkins University School of Medicine, Baltimore, MD 21287, USA; tpurvis2@jhmi.edu (T.E.P.); dlubelski@jhmi.edu (D.L.); 2 Cleveland Clinic Center for Spine Health, Cleveland Clinic, 9500 Euclid Ave, S-80, Cleveland, OH 44195, USA; 3 Department of Neurological Surgery, Cleveland Clinic, 9500 Euclid Ave, S-40, Cleveland, OH 44195, USA

**Keywords:** demyelinating disease, multiple sclerosis (MS), cervical spondylotic myelopathy (CSM), Parkinson’s disease (PD), demyelination, myelopathy, outcomes

## Abstract

A subset of patients with a demyelinating disease suffer from concurrent cervical spondylotic myelopathy, both of which evince similar symptomatology. Differentiating the cause of these symptoms is challenging, and little research has been done on patients with coexisting diseases. This review explores the current literature on the appropriate surgical management of patients with concurrent multiple sclerosis (MS) and cervical spondylotic myelopathy (CSM), and those with both Parkinson’s disease (PD) and CSM. MS and CSM patients may benefit from surgery to reduce pain and radiculopathy. Surgical management in PD and CSM patients has shown minimal quality-of-life improvement. Future studies are needed to better characterize demyelinating disease patients with concurrent disease and to determine ideal medical or surgical treatment.

## 1. Introduction

Demyelinating diseases commonly present symptoms such as muscle weakness, stiffness and spasms, gait disorders, pain, changes in sensation, and disruptions in bowel and bladder function [1,2]. While the pathophysiology of multiple sclerosis (MS) and cervical spondylotic myelopathy (CSM) differs—MS via an autoimmune process and CSM by a mechanical compressive process—both are characterized by damage to myelin and have overlapping presentations [3,4]. Coexisting disorders such as Parkinson’s disease (PD) and CSM can also cause similar symptoms that create difficulty when attempting to differentiate the diseases for treatment or monitoring purposes [2,5,6,7]. The primary objective of decompression and fusion in treatment of CSM is to prevent progression of neurological decline. In many patients, however, there may be improvement in patients’ symptoms and functional status [8]. Little is known about the clinical and quality-of-life (QOL) outcomes following spine surgery for cervical myelopathy in patients with a coexistent demyelinating disease with similar symptoms. This review article seeks to describe such surgical outcomes reported in the literature for patients with concurrent MS and CSM and concurrent PD and CSM.

## 2. Materials and Methods

### 2.1. Search Strategy

A review of the literature was performed using the US National Library of Medicine PubMed database and a hand-search strategy to identify references from the selected articles. The search query included the following terms: demyelinating disease, multiple sclerosis (MS), cervical spondylotic myelopathy (CSM), Parkinson’s disease (PD), amyotrophic lateral sclerosis (ALS), demyelination, and myelopathy.

### 2.2. Eligibility Criteria

Studies were included if they were written in English or had an English translation, and the patient population was comprised of those with a demyelinating disease and coexisting CSM. 

## 3. Results

A total of nine studies were identified that met the inclusion criteria, including eight with concurrent MS and CSM and one with PD and CSM. The identified studies were case reports or case series (Table 1). No prospective studies were identified.

### 3.1. Concurrent Multiple Sclerosis and Cervical Spondylotic Myelopathy

MS is a progressive autoimmune demyelinating disease that affects approximately 0.1% of the United States population [2,18,19,20,21]. MS can occur together with CSM and, although the incidence of concurrent disease has not been reported, is understood to occur. The symptoms are similar for both diseases, including bowel and bladder dysfunction, spasticity, gait ataxia, and sensory deficits [2]. Treatment for the two conditions differs greatly, as the pathophysiology of the myelopathy is very different. Typically, progressive or advanced CSM is treated with surgical decompression [2,18,19] whereas MS is managed medically with corticosteroids or interferon beta [21,22]. Little is known about the surgical or QOL outcomes in concurrent MS and CSM patients treated with spine surgery.

#### 3.1.1. Surgical Outcomes in Patients with Concurrent MS and CSM

In a 1957 report on patients with coexisting MS and cervical spondylosis, Brain and Wilkinson [9] described 17 patients and the challenges that arose in diagnosis and treatment for both diseases. The authors described poor outcomes following laminectomy, particularly for patients with disseminated sclerosis. Given the progressive nature of MS, the authors recommended against any operation that would provide only transitory relief and instead suggested neck immobilization in a collar as a treatment alternative. The authors recognized that for patients who do not have MS, however, a collar may provide suboptimal relief of the spondylosis.

More recent studies have demonstrated conflicting information that instead shows the potential benefits of surgery in patients with MS and CSM. In a study of seven patients with concurrent disease, Young and colleagues [10] found that five patients treated with decompressive surgery showed postoperative improvement in spondylosis symptoms (mean follow-up, 14 months; range, 6–24 months). One patient developed acute MS symptoms a day after surgery. The authors concluded that surgical treatment of spondylosis in patients with coexisting MS and CSM improves symptoms and that MS flare following surgery is rare. 

Arnold et al. [11] came to similar conclusions in a case series of 15 patients with MS and cervical myeloradiculopathy who were treated with surgical decompression and fusion (mean follow-up, 47 months). Thirteen patients demonstrated improvement in upper and lower extremity strength and neck and/or upper extremity pain or paresthesias. In the remaining two patients, symptoms did not improve but did not worsen either. No surgical complications were reported. The authors concluded that surgical intervention for cervical myeloradiculopathy should be considered a safe and effective option in patients with concurrent MS.

One study by Burgerman and colleagues [12] suggested that not all forms of surgical treatment may be effective in patients with coexistent MS and CSM. In a series of six patients, surgery resulted in lasting improvement of symptoms in two of three patients who underwent anterior cervical discectomy (mean follow-up, 30 months; range, 12 months–6 years). One patient treated with cervical laminectomy showed only transient clinical improvement, while three patients (two laminectomies, one anterior cervical discectomy) showed no change in symptoms. The authors suggested that patients who develop progressively worse anatomic compression should be evaluated for surgical treatment. 

In a larger retrospective review of 77 patients with concurrent MS and CSM that were matched with 77 patients with only CSM, all of whom underwent cervical decompression surgery, Lubelski et al. [13] reported that both populations had postoperative improvement. MS and control patients were followed for an average of 58 months and 49 months, respectively. Patients with concurrent MS and CSM had improvements that were less dramatic than those in the control group. A significantly greater proportion of patients in the MS group had myelopathic symptoms that did not improve with surgery in both the short-term (39% in the MS group did not improve vs. 23% in the control group; *p* = 0.04) and long-term (44% in the MS group did not improve vs. 19% in the control group; *p* = 0.004). Patients with primary and secondary progressive MS did show poorer outcomes compared to patients with relapsing remitting MS. Both controls and patients with coexisting MS and CSM had similar postoperative improvement in neck pain and radicular symptoms. The authors concluded that surgery can be recommended to MS and CSM patients, although they should be advised of the potential for less relief of myelopathic symptoms than if they had CSM alone. 

Bashir et al. [14] published a case series that found similar outcomes in patients with MS and coexisting spinal cord compression due to cervical spondylosis or cervical disc disease. Fourteen patients underwent cervical decompression surgery to address presenting symptoms of neck pain (*n* = 11), cervical radiculopathy (*n* = 10), and progressive myelopathy (*n* = 13) (mean follow-up, 3.8 years; range, 1.0–9.8 years). All patients with neck pain reported improvement in or elimination of their pain (*n* = 11). Six of the 10 patients with cervical radiculopathy reported complete resolution of their radicular symptoms, and four reported a reduction. Seven of the 13 patients with progressive myelopathy experienced no improvement in symptoms, although this group uniformly had improvement in or elimination of radicular complaints and neck pain. These results are consistent with those of Lubelski et al. [13], that demonstrated improvement in neck and radicular pain in MS and CSM patients. 

One study by Tan and colleagues [15] did show a reduction in myelopathy in addition to an improvement in radicular symptoms and neck pain. Eighteen patients with concurrent MS and CSM were identified after undergoing cervical spine decompression and fusion (mean follow-up, 18 months; range, 3–45 months). The severity of MS symptoms was assessed using the Expanded Disability Status Scale (EDSS). Of the 14 patients with preoperative myelopathy, four reported improvement (28.6%), nine (64.3%) reported stabilization, and one (7.1%) described a worsening of myelopathy postoperatively. All seven patients with neck pain described elimination of or significant improvement in symptoms. Improvement of radiculopathy occurred in four of five patients (80%) who had preoperative symptoms. No patients with preoperative bladder dysfunction (*n* = 8) experienced relief following surgery. EDSS scores in 16 patients decreased or stabilized (94.4%), while scores increased in two patients (5.6%). The authors explained that their findings were consistent with those of Lubelski et al. [13] in that most patients with myelopathy achieved only stability in symptoms (62%) rather than improvement (30%). These results, together with those of Young et al. [10], Arnold et al. [11], Burgerman et al. [12], Lubelski et al. [13], and Bashir et al. [14] reported above, suggest that surgical treatment may be indicated for relief of neck pain and radicular symptoms rather than the myelopathic symptoms that will progress with MS. Moreover, the collective evidence suggests that surgery does not result in exacerbations of MS. Finally, although MS would likely demonstrate periods of remission in the most common relapsing/remitting variant [23], CSM would otherwise have continuous and progressive myelopathic symptoms.

#### 3.1.2. Quality-of-Life Outcomes in Patients with Concurrent MS and CSM

While surgical outcomes such as neurological status and complications have been investigated in patients with coexisting MS and CSM, only one study has examined the QOL outcomes in these patients with concurrent disease. Lubelski et al. [16] identified 13 patients with MS and CSM and 52 control patients with CSM alone who were treated with cervical decompression (mean follow-up was 22 and 18 months, respectively). QOL was assessed using the EuroQol 5-Dimensions (EQ-5D) metric that includes the domains of anxiety/depression, usual activities, self-care, mobility, and pain/discomfort. Patients in the control group had significantly improved QOL scores in three domains (mobility, *p* = 0.04; self-care, 0.003; anxiety/depression, *p* = 0.03), measured from pre- to post-operative status, in contrast to patients with concurrent disease. Quality-Adjusted Life-Year (QALY) measurements, or the years of life added as a result of the surgery, in the concurrent MS and CSM group did not change significantly from pre- to post-operation (*p* = 0.96), while those in the control CSM group had a significant change from a QALY of 0.50 to 0.64 (*p* < 0.0001). Only the CSM controls showed a change in QALY that was greater than the minimal clinically important difference (MCID) of 0.1. A majority of patients with CSM and MS did, however, experience improvement in QALY (54%). These results suggest that while surgery may still be indicated for patients with concurrent disease, patients may not experience QOL benefits following the intervention despite an improvement in pain, radicular symptoms, and potentially myelopathy. 

These studies demonstrate that MS and CSM have symptoms that are overlapping, making it difficult to correctly attribute any one symptom to the appropriate causative disease entity. The progressive myelopathic symptoms of CSM, as well as the potential benefit of surgery in relieving pain and radicular symptoms, may warrant surgical intervention in patients with concurrent disease. However, outcomes may be suboptimal in these patients compared to those with CSM alone. Patients should be appropriately educated about the potential impact of MS on their surgical outcomes. 

### 3.2. Concurrent Parkinson’s Disease and Cervical Spondylotic Myelopathy

PD affects approximately 1% of individuals over the age of 60 [24,25]. Symptoms of PD are many and diverse, and include tremor, weakness, a variety of movement disorders (e.g., ataxia, shuffling gait, involuntary movements, motor retardation), and bladder or bowel dysfunction [5,6,7,17]. CSM is characterized by similar symptoms [26], and distinguishing between the two pathologies in patients with coexistent diseases can be challenging. Treatment of CSM is most commonly surgical decompression and fusion, which leads to improvement in QOL [27,28,29,30,31,32,33,34]. Among patients with PD, however, spine surgery can be associated with poor post-operative QOL and may lead to high complication and reoperation rates [35,36,37,38,39,40]. Treatment of PD is typically pharmacologic or, if necessary, deep brain stimulation [41,42,43,44,45,46]. Untreated CSM, however, is also associated with worsening symptoms and QOL, and accordingly the question arises as to how best treat patients with concurrent PD and CSM. 

Research on patient populations with concurrent PD and CSM is scant. The first study in this population examined QOL outcomes following cervical decompression [17]. Xiao et al. [17] performed a retrospective matched cohort analysis that included 11 patients with PD and CSM matched to 44 controls with CSM alone who underwent cervical decompression (mean follow-up was 12.4 and 13.4 months, respectively). QOL was assessed using several patient-reported health status measurements, including the EQ-5D, Pain Disability Questionnaire (PDQ), and Patient Health Questionnaire-9 (PHQ-9). Patients with concurrent PD and CSM demonstrated a statistically significant reduction in postoperative pain-related disability. However, these changes were less substantial than in control patients. Although PD patients and controls had similar preoperative QOL scores, a smaller proportion of PD patients obtained an MCID in EQ-5D (18% vs. 57%, *p* = 0.04). Upon the last follow-up visit, PD patients also reported worse QOL as measured by EQ-5D (0.526 vs. 0.707, *p* = 0.01) and PDQ (80.7 vs. 51.4, *p* = 0.03). PD was an independent risk factor for a smaller improvement in EQ-5D scores (β = −0.09, *p* < 0.01) and an inability to obtain an MCID in EQ-5D scores (odds ratio: 0.08, *p* < 0.01). The proportion of patients achieving an MCID in PHQ-9 or PDQ scores was not significantly different between groups.

These results suggest that cervical decompression has minimal benefit in a patient population with coexisting PD and CSM. While spine surgery may provide some reduction in pain-related disability, QOL outcomes were poor compared to controls. In this patient population, preoperative counseling of risks and benefits is integral. And while surgery will provide some benefit, it will certainly not be as great as it could be for those with only CSM. Ultimately, the natural history of PD will lead to progressive worsening in symptoms over time. Of note, the small sample size of this study may not achieve adequate power to detect an effect. Future studies with larger numbers of PD and CSM patients are necessary to confirm the findings of Xiao et al. [17].

## 4. Limitations

This review is limited by the small sample sizes and retrospective nature of the studies included. Surgical outcome measures were not standardized among studies, which reduces their comparability. Selection of inappropriate surgical candidates or differing surgical skill may also have affected success rates. Moreover, the method of diagnosis of CSM was not standardized among the included studies, and this may have led to conflicting findings. Lastly, radiological interpretation by radiologists may result in reporting of non-essential or incidental findings that suggest surgical intervention in patients who may not otherwise have been identified by surgeons’ radiological interpretations. Surgical approach during decompression also differed among studies, further limiting comparability. 

## 5. Conclusions

While the primary goal of surgical intervention for CSM may remain prevention of progressive neurological decline, surgery also has the potential for symptomatic and quality-of-life improvement. There exists conflicting information about the success of spine surgery in reducing symptoms in MS and CSM patients, but most recent research suggests that surgery reduces preoperative pain, radicular symptoms, and possibly myelopathy. The improvement, however, is less than in those without MS. In patients with coexisting PD and CSM, surgical management may reduce some axial and radicular pain symptoms but results in QOL outcomes that may not be clinically significant. These findings suggest that surgery reduces clinical symptoms in these populations with concurrent diseases but that the outcomes will not be as good as in those patients with CSM alone. While studies indicate surgical intervention in patients with coexistent diseases (CSM/PD, CSM/MS) results in less favorable outcomes when compared to CSM alone, the authors believe that the former patient population perhaps has more to lose if compressive myelopathy is left untreated given the smaller functional margin at baseline. It is important that a rational and multispecialty approach (spine surgeons, internists and neurologists, and patient) be taken when constructing a treatment plan for this delicate patient population. Future research is needed in these unique patient populations to determine optimal treatment and to better predict for which patients surgery may provide symptomatic relief. Moreover, appropriately counseling patients with concurrent diseases, especially with regards to the natural course of the disease, is crucial. 

## Figures and Tables

**Table 1:** Reviewed literature on demyelinating disease and coexisting disease with similar symptoms.

Authors	Year	Number of Patients	Surgical Intervention	Mean Follow-Up Time (Months)	Main Study Findings
Concurrent Multiple Sclerosis and Cervical Spondylotic Myelopathy					
Surgical Outcomes in Patients with Concurrent MS and CSM					
Brain and Wilkinson [9]	1957	17 with MS and CSM	Laminectomy	--------	Patients reported poor outcomes following laminectomy, particularly for those with disseminated sclerosis.
Young et al. [10]	1999	7 with MS and CSM	Decompression	14 (range 6–24)	5 patients showed postoperative improvement in spondylosis symptoms. 1 patient developed acute MS symptoms a day after surgery.
Arnold et al. [11]	2011	15 with MS and cervical myeloradiculopathy	Decompression, fusion, and fixation	47	13 patients demonstrated objective improvement in upper and lower extremity strength and neck and/or upper extremity pain or paresthesias.
Burgerman et al. [12]	1992	6 with MS and CSM	Anterior cervical discectomy or cervical laminectomy	30 (12–72)	Long-term improvement in 2/3 patients with anterior cervical discectomy. 1 patient treated with cervical laminectomy showed only transient clinical improvement. 3 patients (2 laminectomies, 1 anterior cervical discectomy) showed no change in symptoms.
Lubelski et al. [13]	2014	77 with MS and CSM; 77 with CSM	Cervical decompression	57.7 ± 43.3 (MS and CSM); 49.4 ± 42.5 (CSM)	39% in the MS group did not have myelopathy improvement in the short-term vs. 23% in the control group (*p* = 0.04) and, in the long-term, 44% in the MS group did not improve vs. 19% in the control group (*p* = 0.004).
Bashir et al. [14]	2000	14 with MS and spinal cord compression	Cervical decompression	45.6 (range, 12.0–117.6)	All patients with neck pain reported improvement in or elimination of their pain (*n* = 11). 6/10 patients with cervical radiculopathy reported complete resolution of their radicular symptoms, and 4 reported a reduction. 7/13 patients with progressive myelopathy experienced no improvement in symptoms.
Tan et al. [15]	2014	18 with MS and CSM	Cervical decompression and fusion	18 (range, 3–45)	4 reported improvement (28.6%), 9 (64.3%) reported stabilization, and 1 (7.1%) described a worsening of myelopathy. All 7 patients with neck pain described elimination of or significant improvement in symptoms.
Quality-of-Life Outcomes in Patients with Concurrent MS and CSM					
Lubelski et al. [16]	2014	13 with MS and CSM; 52 controls with CSM	Cervical decompression	22.3 ± 10.6 (MS and CSM); 18.2 ± 10.8 (CSM)	QALY in the MS and CSM group did not change significantly from pre- to post-operation (*p* = 0.96) vs. a significant change in the control CSM group from a QALY of 0.50 to 0.64 (*p* < 0.0001).
Concurrent Parkinson’s Disease and Cervical Spondylotic Myelopathy					
Xiao et al. [17]	2016	11 with PD and CSM; 44 controls with CSM	Cervical decompression	12.4 ± 16.2 (PD and CSM); 13.4 ± 11.3 (CSM)	Patients with PD and CSM reported worse quality-of-life at last follow-up than controls (0.526 vs. 0.707, *p* = 0.01). PD and CSM patients did have improvement in pain-related disability.

PD: Parkinson’s Disease; CSM: Cervical Spondylotic Myelopathy; MS: Multiple Sclerosis; QALY: Quality-Adjusted Life-Year.
